# Persistent *Bacillus cereus* Bacteremia in 3 Persons Who Inject Drugs, San Diego, California, USA

**DOI:** 10.3201/eid2209.150647

**Published:** 2016-09

**Authors:** Gabrielle Schaefer, Wesley Campbell, Jeffrey Jenks, Cari Beesley, Theodoros Katsivas, Alex Hoffmaster, Sanjay R. Mehta, Sharon Reed

**Affiliations:** UC San Diego Health, San Diego, California, USA (G. Schaefer, J. Jenks, T. Katsivas, S.R. Mehta, S. Reed);; Naval Medical Center San Diego, San Diego (W. Campbell);; Centers for Disease Control and Prevention, Atlanta, Georgia, USA (C. Beesley, A. Hoffmaster);; San Diego Veterans Affairs Medical Center, San Diego (S.R. Mehta)

**Keywords:** bacillus, Bacillus cereus, bacteremia, California, injection drug use, heroin, persons who inject drugs, multilocus sequence typing, bacteria, United States

## Abstract

*Bacillus cereus* is typically considered a blood culture contaminant; however, its presence in blood cultures can indicate true bacteremia. We report 4 episodes of *B. cereus* bacteremia in 3 persons who inject drugs. Multilocus sequence typing showed that the temporally associated infections were caused by unrelated clones.

*Bacillus* species are typically considered blood culture contaminants, and distinguishing true versus pseudobacteremia requires recognition of the clinical context. Risk factors for infection include prosthetic heart valves, pacemakers, injection drug use, and immunosuppression (*1*). In 2013 in San Diego, California, USA, 3 persons who inject drugs (PWIDs) were diagnosed with persistent *B. cereus* bacteremia. To determine if there was a common source of infection, we performed multilocus sequence typing (MLST) of *B. cereus *from these patients.

## The Study

In September 2013, a 19-year-old woman (patient 1) sought care in the emergency department (ED) of the UC San Diego Medical Center, reporting a 2-day history of headache, fever, myalgia, nausea, vomiting, diarrhea, and right upper quadrant pain. Two days earlier she had injected heroin, using a clean needle and a previously used cotton filter. At admission, she had a fever (100°F), increased heart rate (112 beats/min), and right upper quadrant tenderness but no evidence of endocarditis. Test results showed leukocytosis (26.0 × 10^9 ^cells/L) and elevated levels of aspartate aminotransferase (107 U/L), alanine aminotransferase (112 U/L), and C-reactive protein (CRP; 6.5 mg/dL). Results for rapid HIV antibody test, acute hepatitis panel, and urine pregnancy test were negative. Intravenous (IV) fluids and empiric IV vancomycin and piperacillin/tazobactam were administered; symptoms and clinical status were improved the next day. Cultures (4/4) of blood samples collected at admission grew *B. cereus* sensitive to clindamycin, gentamicin, and vancomycin. Transthoracic echocardiography showed no abnormalities.

Over the next 2 days, she remained afebrile and symptoms resolved, but bacteremia persisted. On hospital day 5, given the persistent bacteremia, we stopped piperacillin/tazobactam and started IV ciprofloxacin. Gentamicin was added on days 8–12 for its potential synergistic effect; bacteremia resolved on day 9. IV vancomycin and ciprofloxacin were continued for 4 weeks.

Fifteen days after patient 1 was admitted, a 43-year-old man (patient 2) sought care at the same institution for low back pain. He had used heroin 2 days earlier; injection technique details are unknown. At admission, he was afebrile and had vital signs and blood cell counts within the normal range, no focal neurologic findings, and unrevealing magnetic resonance imaging results. The patient was discharged with a diagnosis of musculoskeletal back pain. After *B. cereus* grew in 1 of 2 blood cultures, he was asked to return. Although asymptomatic and lacking any stigmata of endocarditis, he was admitted for further observation and started empirically on IV vancomycin. Blood cultures continued to grow clindamycin- and vancomycin-sensitive *B. cereus* until hospital day 4. Transesophageal echocardiography showed no evidence of endocarditis. IV vancomycin was continued for 2 weeks.

In October 2013, a 58-year-old man (patient 3) sought care in the ED of an affiliated hospital, the VA San Diego Healthcare System, for debilitating abdominal pain. He had a prosthetic right hip and history of chronic polyarticular pain. He admitted previous injection of crushed oxycodone but denied recent injections. Laboratory results showed leukocytosis (21.2 × 10^9^ cells/L) and a high CRP level (36 mg/dL). Abdominal computed tomography imaging showed circumferential ileal wall thickening, and endoscopy showed bleeding and friable ulcerations. Blood cultures (6/8) grew gram-positive bacilli. Vancomycin and piperacillin/tazobactam were initiated, but piperacillin/tazobactam was changed to oral levofloxacin once *B. cereus* was identified. Transthoracic echocardiography showed a nonmobile aortic valve vegetation, suggesting endocarditis. Blood cultures remained *B. cereus*–positive over 6 days of therapy, so gentamicin was added for 7 days, followed by 6 weeks’ treatment with only vancomycin and levofloxacin. Crohn disease was diagnosed based on imaging findings.

In January 2015, patient 3 returned to the ED for a pulmonary syndrome, which was diagnosed as bronchitis and treated with azithromycin. Blood sample cultures (2/2) grew *B. cereus*. The patient was afebrile and had no clinical findings. His leukocyte count was within reference range, and CRP was marginally elevated (0.77 mg/dL). He admitted to resuming injection of crushed oxycodone with unused paraphernalia because of increasing right hip pain. Transthoracic echocardiography at admission showed no evidence of the previous vegetation or any new lesions, and results of a tagged leukocyte scan were negative, suggesting that neither the prior vegetation nor the prosthetic hip was the source of the new bacteremia. After 7 days of observation and multiple negative blood cultures, the patient was discharged without antimicrobial drug therapy. However, 1 of 4 cultures of blood samples obtained on the day of discharge grew *B. cereus*, so he returned to the ED, where physical examination showed no abnormalities and laboratory test results were negative. The patient refused admission and was discharged on a 6-week course of oral ciprofloxacin with outpatient follow-up. He denied use of injection drugs while an inpatient or after discharge.

To determine if isolates from the 3 patients were related, we performed MLST to characterize 7 alleles and their phylogenetic lineage based on sequence type (*2*,*3*), using the *B. cereus* MLST website (http://pubmlst.org/bcereus/). Isolates from each patient were genetically distinct; however, the 2 isolates from the most recent admission of patient 3 were clonal but different than his 2013 isolate (Table). These findings reinforced the presumption of a true bacteremia and that a prolonged course of antimicrobial drugs was warranted for patient 3. The persistent bacteremia from 2013 and the clonal isolates from 2015 demonstrate 2 unique episodes of infection probably associated with injection of crushed oxycodone as the common risk factor.

**Table:** Multilocus sequence typing results for *Bacillus cereus* isolates from 3 persons who inject drugs, San Diego, California, USA

Patient no.	Alleles							Sequence type
	*glpF*	*gmk*	*ilvD*	*pta*	*pur*	*pycA*	*tpi*	
1	13	8	46	28	9	12	7	100
2	105	61	95	92	92	77	68	247
3								
2013 isolate	172	71	14	29	24	14	7	787
2015 admission isolate	12	8	9	14	11	12	10	24
2015 discharge isolate	12	8	9	14	11	12	10	24

We identified 4 episodes of *B. cereus* bacteremia in 3 PWIDs. Although often considered a blood culture contaminant, *B. cereus* can indicate true bacteremia in the appropriate clinical context. *B. cereus* bacteremia has previously been reported in PWIDs (*4*), and cultures of heroin samples and injection drug use paraphernalia have been predominantly positive for *Bacillus* species (*4*–*6*). In another study, spore-forming bacteria (*B. cereus, B. anthracis,* *Clostridium botulinum*) were shown to contaminate heroin (*7*). Endocarditis caused by *B. cereus* has also been documented in PWIDs (*8*).

A 2014 epidemiologic review of *B. cereus* bloodstream infections identified 51 cases meeting Infectious Disease Society of America guidelines for true bacteremia (*9*). The review showed increased rates of environmental isolation of *B. cereus* in summer, possibly due to a higher environmental load of *B. cereus* during this season. In line with our clinical experience, the findings from that single-center study showed no clonality, supporting the assumption that contamination may be multiclonal (*9*). Given that multiclonality would be identified in the clinical setting only if colonies differed in appearance, it is possible that the reported cases of bacteremia might also have been multiclonal.

Although there are no established treatment guidelines, most *B. cereus* species produce β-lactamases and thus show resistance to β-lactam antimicrobial drugs. Sensitivity to other antimicrobial drugs has been studied, but variation in in vitro methods demonstrates inconsistencies with susceptibility cutoffs (*10*). *B. cereus* is considered susceptible to aminoglycosides, carbapenems, and fluoroquinolones, and newer antimicrobial drugs (linezolid, daptomycin, telavancin) may have activity against *B. cereus* (*10*–*12*); however, the true effect of the in vitro MICs on clinical outcomes is unclear. To achieve clearance of bacteremia, 2 of the 3 patients in our study required the addition of gentamicin (an aminoglycoside) to their treatment regimen. At follow-up, all 3 patients were clinically well without recurrence of bacteremia.

## Conclusions

Identification of genetically distinct isolates of *B. cereus* in these temporally associated cases is consistent with findings from previous work and highlights the environment as the source of these cases of bacteremia. However, given the potential for *B. cereus* to cause serious disease, isolation of this organism should prompt a complete clinical evaluation.
